# Trends In The Alignment And Harmonization Of Reproductive, Maternal, Newborn, And Child Health Funding, 2008–13

**DOI:** 10.1377/hlthaff.2017.0364

**Published:** 2017-11-01

**Authors:** Melisa Martinez-Alvarez, Arnab Acharya, Leonardo Arregoces, Lara Brearley, Catherine Pitt, Christopher Grollman, Josephine Borghi

**Affiliations:** 1 Assistant professor in the Department of Global Health and Development, London School of Hygiene and Tropical Medicine, in the United Kingdom; 2 Honorary associate professor in the Department of Global Health and Development, London School of Hygiene and Tropical Medicine; 3 Research degree student in the Department of Global Health and Development, London School of Hygiene and Tropical Medicine; 4 Senior health policy adviser at Save the Children in London; 5 Assistant professor of health economics in the Department of Global Health and Development, London School of Hygiene and Tropical Medicine; 6 Research fellow in the Department of Global Health and Development, London School of Hygiene and Tropical Medicine; 7 Associate professor of health economics and policy in the Department of Global Health and Development, London School of Hygiene and Tropical Medicine

## Abstract

Donor financing to low- and middle-income countries for reproductive, maternal, newborn, and child health increased substantially from 2008 to 2013. However, increased spending by donors might not improve outcomes, if funds are delivered in ways that undermine countries’ public financial management systems and incur high transaction costs for project implementation. We combined quantitative and qualitative methods to examine the quality of funding for reproductive, maternal, newborn, and child health globally and in Tanzania, based on two principles of aid effectiveness: the alignment of donor financing with the recipient country’s public health financial management systems, and donor harmonization for coordinated, transparent, and collectively effective actions. We found that alignment of donor financing deteriorated throughout the period, with the proportion of funds channeled through governments decreasing from 47 percent to 39 percent. Tanzania-based donors attributed the change to the pressure donors were under to achieve and show results. Donor harmonization was low overall and remained relatively constant, although it increased in sub-Saharan Africa and decreased in South Asia. Bilateral funding agencies were the most harmonized donors. We recommend that future assessments of Sustainable Development Goals financing include measures of harmonization and alignment of funding.

Oficial development assistance for health from wealthy countries to low- and middle-income countries quadrupled from $5 billion in 1990 to over $21 billion in 2013.^1^ The increase was accompanied by an expansion of actors and initiatives in the health sector, including global health initiatives. Simultaneously, interest in the effectiveness of official development assistance has grown, a topic addressed in a series of high-level forums and international conferences—in Monterrey, Mexico (2002); Rome, Italy (2003); Paris, France (2005); Accra, Ghana (2008); Busan, South Korea (2011); Mexico City (2014); and Addis Ababa, Ethiopia (2015)—each of which issued declarations. The Paris declaration articulated five principles of aid effectiveness: country ownership of national strategies; alignment of aid with country strategies; harmonization, or coordination, of donor aid; results for funding; and accountability between donors and aid recipients.^2^ These principles encourage providers of official development assistance to align their funding with a recipient country’s development strategies and systems, so that donors’ activities are harmonized, and recipients and donors focus on achieving results for which they are mutually accountable.^2^ These principles form the core of the declarations mentioned above and have been collectively defined as the “global aid effectiveness agenda.”^3^

Most of the literature to date on official development assistance for health has focused on tracking its distribution from donors to countries and its targeting to countries’ needs.^4–8^ Less attention has been paid to its effectiveness in relation to the Paris principles, although there is evidence that funding fragmentation,^9^ volatility,^10^ and high transaction costs for recipient governments^11^ limit both the impact of official development assistance on health and the sustainability of progress already achieved. A 2014 report found progress in the use of country-results frameworks and joint assessments of national strategies, but it reported reduced use of national financial management procedures and less predictable funding for 2015–17.^12^ There have been further concerns that the focus on achieving global goals (including the Millennium Development Goals of the United Nations) and targets based on national averages have incentivized programs to focus on easily attained targets, thereby widening inequities^13^ and favoring specific health conditions and population groups.^14,15^ In addition, little attention has been paid to the adherence to aid effectiveness principles of health donors that target specific populations or diseases—despite recognition of the fact that providing assistance in the form of vertical projects (that is, funds designated for specific diseases or population groups) contributes to the proliferation of programs, fragmentation of programming, and transaction costs for national health ministries and hinders donor harmonization.^16,17^

We used a mixed-methods approach to assess whether there were improvements in the alignment and harmonization of donor funding for reproductive, maternal, newborn, and child health between 2008 and 2013, both at the global level for donors and recipients and at the country level, using Tanzania as a case study. We focused on this funding because of the large increase in donor funding in recent years to low- and middle-income countries related to Millennium Development Goals 4 and 5 (to improve child survival and maternal health, respectively).^18^ We used a case study to highlight how global trends affect national ministries and country-based donors. We selected Tanzania as our case study because it is a low-income recipient country that has a high degree of dependency on official development assistance and that experienced a substantial increase in external reproductive, maternal, newborn, and child health funding between 2008 and 2013.^18^ It is also a country in which we have extensive experience working and living, and we are therefore familiar with its health systems and relevant stakeholders.

## Study Data And Methods

**Data Sources** Quantitative data for both global and country-level analyses were extracted for the period 2008–13 from the Countdown ODA+ data set, which tracks flows of official development assistance (ODA) and private funds from the Bill and Melinda Gates Foundation (collectively referred to hereafter as ODA+).^18,19^ The data set includes information about sixty-four donors and 147 recipient countries and is based on the Organization for Economic Cooperation and Development’s (OECD’s) Creditor Reporting System (CRS) database, to which it applies the Countdown project classification for reproductive, maternal, newborn, and child health (RMNCH).^18^ All records in the CRS are individually classified as RMNCH following the Countdown framework^18^—in which both the full value of vertical funds (such as those for family planning, emergency obstetric care, and childhood vaccination) and a proportion of the value of funding for primary health care, HIV prevention and treatment, health-sector budget support, general budget support, and so on are considered to promote reproductive, maternal, newborn, and child health.^18^

For the qualitative component of the study, Melisa Martinez-Alvarez conducted semistructured interviews with members of the headquarters staff of four of the top ten donors to reproductive, maternal, newborn, and child health (*n* = 4), representatives of donors in Tanzania (*n* = 7), and representatives of governmental and nongovernmental organizations working in the Tanzanian health sector (*n* = 15). The interviews explored whether and how principles of aid effectiveness are considered in resource allocation and the perceptions of trends in resource allocation patterns over time and their consequences. A semistructured interview tool was used to guide the interviews (see the interview guide in the online Appendix).^20^

**analytical framework** We developed an analytical framework to assess progress toward alignment and harmonization of ODA+ to reproductive, maternal, newborn, and child health, based on the definitions in the Paris Declaration on Aid Effectiveness2 that could be feasibly measured with our data (Appendix Table 1).^20^

According to the Paris Declaration, *alignment* refers to the degree to which donors base their support on recipient countries’ national development strategies, institutions, and procedures.^2^ The Paris indicators for assessing the alignment of donor funding focus on the proportion of official development assistance that uses national financial systems, is reported on national budgets, and is predictable and untied (that is, it can be used to purchase goods and services from any country), as well as the quality of country systems.^2,21^ The Countdown data did not allow us to assess the proportion of funds reported on national budgets or the degree of tying of ODA+ to reproductive, maternal, newborn, and child health. Instead, we examined the proportion of funds disbursed through government systems and the proportion of funds that were pooled, to assess the use of national financial systems; and the volatility of funds from the top ten donors of ODA+ for reproductive, maternal, newborn, and child health at the country level in Tanzania as a proxy for predictability.

Tanzania received ODA+ for this area of health from twenty-eight to thirty-two donors during the study period. The top ten donors accounted for 88.2 percent of the funds (and therefore would have had the most impact on the volatility of funding); we discuss only these funders in our analysis of volatility. We therefore measured alignment by the share of ODA+ delivered through government channels by donor and by recipient country; the share of ODA+ delivered through the government that used pooled modalities rather than project funding; and volatility in total ODA+ to reproductive, maternal, newborn, and child health disbursed by donors to Tanzania.^22^

*Harmonization* is defined in the Paris Declaration as the degree to which donors’ actions are coordinated, transparent, and collectively effective.^2^ The indicators used to evaluate harmonization according to that definition are the use of common arrangements and procedures and shared analysis.^2,21^ Our data did not allow us to systematically compare donors and recipient countries using these indicators. Furthermore, we did not consider them to be suitable ways to assess the impact of donor activities on recipient countries. Instead, we assessed the fragmentation and proliferation of funding for reproductive, maternal, newborn, and child health, which donors committed to reduce in the Paris^2^ and Accra Declarations. We did this at the global level through indices of dispersion by assessing donor proliferation and recipient-country fragmentation of funding. A donor is a high proliferator if it distributes its budget among many recipients and a low proliferator if it concentrates its budget among a small number of countries.^11^ *Fragmentation* refers to the number of donors in a given recipient country relative to the total ODA+ to reproductive, maternal, newborn, and child health. A country is highly fragmented if there are many donors, each of which provides a small share of the total ODA+.^11^

**Projects delivered outside the government are the least coordinated with or aligned to country strategies.**

**data analysis** We classified ODA+ to reproductive, maternal, newborn, and child health as disbursed through the government if the OECD’s Creditor Reporting System database classified it as such (CRS channel codes 10000–19999) or if the channel code was empty but the CRS aid type was general budget support (A01) or health-sector budget support (A02). We manually classified projects as disbursed *through the government* if the “channel name” field indicated a government agency, and as *outside the government* if the same field indicated a nongovernmental organization. We considered ODA+ to reproductive, maternal, newborn, and child health to have been delivered as pooled funds if the type of assistance was general budget support (CRS type A01), health-sector budget support (CRS type A02), or basket funds or pooled funding (CRS type B04) explicitly channeled through the government. We analyzed trends in aid type for the period 2009–13, because data for 2008 were incomplete.

**global analysis** We examined donor proliferation and recipient-country fragmentation of funding using the Theil and Herfindahl-Hirschman Indices, respectively^11^ (see the supplementary methods in Appendix),^20^ based on three-year averages of ODA+ to reproductive, maternal, newborn, and child health in the period 2006–11 and the average in the period 2012– 13 to remove yearly variation.^23^ The Theil Index (which ranges from 0 to the natural log of the number of recipients) compares the amount disbursed by a donor to a country to the average amount disbursed by the donor per country. A smaller Theil Index indicates greater proliferation, or that there are many recipients that receive less than the average amount from a particular donor. The Herfindahl-Hirschman Index (HHI) compares the number of donors in a recipient country to the total amount of ODA+ to reproductive, maternal, newborn, and child health that the country receives. It is estimated as a number between 1/*n* and 1, where n is the number of donors disbursing official development assistance to a recipient country. Larger values suggest concentration of ODA+ (in other words, that a single donor or small group of donors contribute a significant share of the assistance); smaller values indicate greater fragmentation in assistance (in other words, the assistance is distributed in small amounts by many donors). Therefore, the larger the HHI, the greater the degree of harmonization of ODA+.

**The findings suggest the need for reflection on the future of official development assistance to low- and middle-income countries.**

We generated measures of alignment and harmonization of funding for every donor and recipient country. We estimated averages across donor types (bilateral, multilateral, global health initiatives, and the Gates Foundation) and recipient country income groups (using the World Bank categories for fiscal year 2018, according to which a 2016 per capita income of $1,005 or less indicated a low-income country, while incomes of $1,006–$3,955, $3,956–$12,235, and $12,236 or more indicated a lower-middle-income country, an upper-middle-income country, and a high-income country, respectively).^24^ We categorized the European Union (EU) as a bilateral donor since EU institutions, rather than member states, ensure coherence and control spending for official development assistance.^25^ We distinguished between multilaterals (made up of multiple members, including UN agencies and Bretton Woods institutions) and global health initiatives (single-issue agencies, including the Global Fund to Fight AIDS, Tuberculosis, and Malaria and GAVI, the Vaccine Alliance). For 2013 we estimated ranges of values for channel, modality, and fragmentation index for each of the income groups (see Appendix Figure 1).^20^ We analyzed fragmentation across geographic regions.

**tanzania case study** To explore the alignment of donor funding with government strategies in Tanzania, we also examined volatility year by year in ODA+ to reproductive, maternal, newborn, and child health disbursements for each of the top ten donors in the period 2008– 13. To measure fragmentation in Tanzania, we calculated the number of donors; the proportion of total ODA+ to reproductive, maternal, newborn, and child health that each donor represented; and the number of transactions for the period 2008–13. We assumed that each record in the CRS database represented a transaction. Although a single project can be delivered as multiple transactions, each transaction incurs costs in terms of reports and meetings.

For the qualitative analysis, interviews were recorded and transcribed. Data from the interviews were analyzed using thematic coding^26^ that was based on the analytical framework (for details, see Appendix Table 1).^20^ The coding framework was developed by Melisa Martinez-Alvarez and Josephine Borghi, and all coding was undertaken by Martinez-Alvarez. NVivo was used to manage the data. Qualitative analysis was undertaken after the analysis of quantitative data. The results of the analyses were integrated during the writing of this article.

**limitations** This study was subject to several methodological limitations. First, we assessed aid effectiveness in relation to the two principles that could be measured with our data. Other important principles of aid effectiveness (country ownership of national strategies, management for results, and mutual accountability of donors and recipients) were not addressed. These principles are difficult to assess across countries, since they require the use of qualitative methods to understand whether the mechanisms in place achieved their intended outcomes.^3^ In addition, we did not determine what the funds were spent on, despite the implications this may have for their effectiveness. Alignment and harmonization of funding could be measured in ways other than those used in this study. For instance, a measure of alignment should consider whether donor funding is filling gaps in national plans, but there is no consistent methodology that can be used to assess this. Similarly, harmonization should be assessed according to the degree to which donors adopt common approaches in recipient countries. This is difficult to achieve across countries. An in-depth case study in Tanzania found that despite coordination mechanisms’ being in place, internal donor structures and incentives were hindering harmonization efforts.^3^ Furthermore, our definition of aid *effectiveness* was restricted to the code of good practice outlined by the global aid effectiveness agenda, instead of being based on an evaluation of the impact of different modalities for ODA+ on reproductive, maternal, newborn, and child health outcomes.

Second, there were some limitations to our data. Information in the Countdown ODA+ database was manually coded by different people in different years, and although the team performed consistency checks, some bias may have still been introduced.^19^ In addition, we made assumptions about the proportions of funds for reproductive, maternal, newborn, and child health that were delivered as general or health-sector budget support, since donors indicated only the funding modality, not its subsequent allocation to reproductive, maternal, newborn, and child health.^18^ In Tanzania, general budget support is disbursed through the Ministry of Finance, so including this type of financing might have resulted in an overestimation of the number of transactions that the Ministry of Health managed. However, we do not anticipate this to be substantial. Our estimates of ODA+ to reproductive, maternal, newborn, and child health differ from those previously reported in Countdown ODA+ analyses^18^ because we excluded funds reported as regional disbursements—since we were interested in aid flows to specific recipient countries. By considering only funds disbursed through the government if that was the channel recorded in the CRS database, we might have underestimated the amount of funds delivered through a third partner but ultimately disbursed to governments (for instance, GAVI disburses 74.3 percent of its ODA+ to reproductive, maternal, newborn, and child health through UNICEF). In addition, we were not able to unpack the drivers of funding allocation. However, this is the subject of work that we are currently conducting.

Finally, only Martinez-Alvarez conducted the interviews and analyzed the qualitative data. This may have biased both the participants’ responses and how they were interpreted.

## Study Results

**alignment of donor funding** In the period 2009–13, 40.9 percent of ODA+ to reproductive, maternal, newborn, and child health was delivered through governments (Exhibit 1). On average, in the period 2008–13, global health initiatives delivered 44.3 percent of their funds through governments; the shares for bilaterals and multilaterals were 38.5 percent and 58.7 percent, respectively (Appendix Table 2).^20^ Of the top ten donors, those channeling the highest proportions of funds through governments were Germany (81.3 percent) and the Global Fund (61.7 percent), and those channeling the lowest proportions were the Gates Foundation (1.4 percent) and GAVI (9.7 percent) (Appendix Table 3).^20^ In the same period, the share of funds for reproductive, maternal, newborn, and child health disbursed through government channels declined from 46.6 percent to 38.7 percent (Exhibit 2). Bilateral agencies and global health initiatives reduced their funding through governments, while the funding of multilaterals increased in 2009 and then stayed constant.

**EXHIBIT 1:**
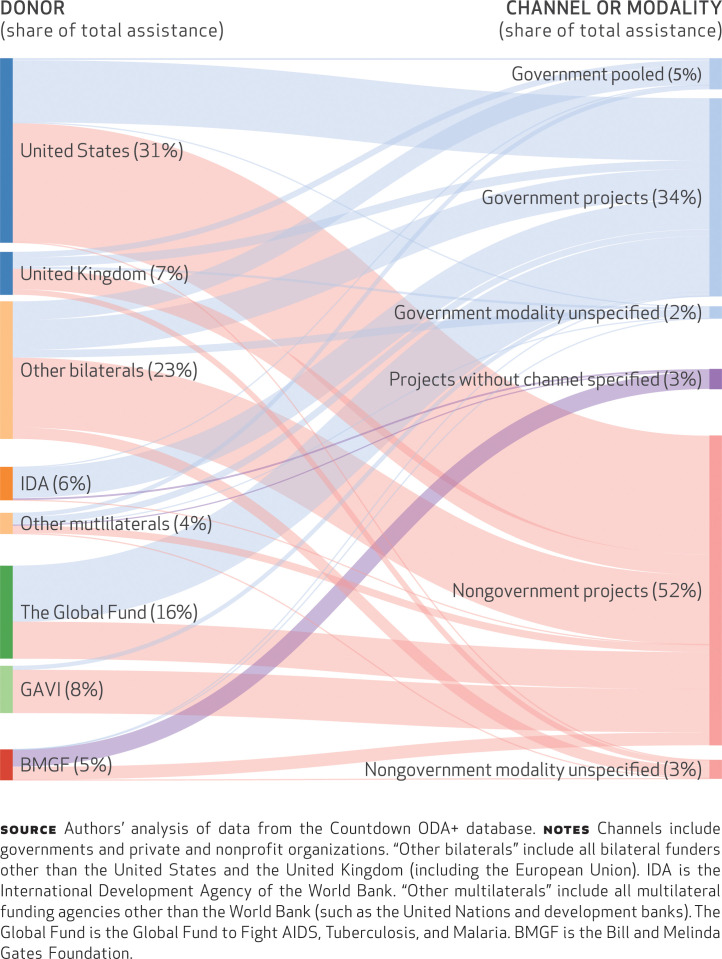
Total official development assistance for reproductive, maternal, newborn, and child health, by donor and channel or modality, 2009–13

**EXHIBIT 2:**
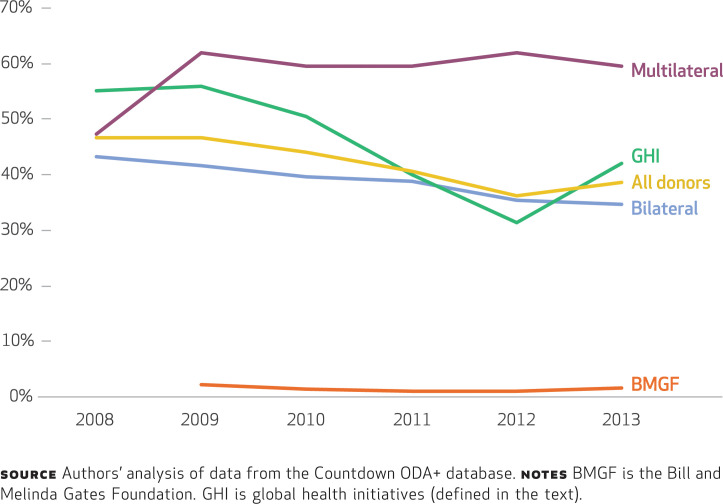
Percentages of official development assistance for reproductive, maternal, newborn, and child health delivered through government channels, by donor type, 2008–13

In the period 2009–13, for those donors delivering ODA+ to reproductive, maternal, newborn, and child health through government channels, only 12.9 percent of the assistance, on average, was delivered as pooled funds with other donors, with 82.0 per cent being disbursed as project funding (there was insufficient information to estimate the modality of the remaining 5.1 percent) (Appendix Table 4).^20^ Of the top ten donors, Canada (86.0 percent), EU institutions (61.7 percent), and the United Kingdom (32.8 percent) disbursed the highest proportion of their funds through governments as pooled funds (Appendix Table 4).^20^ Eighteen donors disbursed none of their funds to governments as pooled funding, including the Global Fund, GAVI, and the Gates Foundation. The share of government funds pooled across all donors increased from 7.5 percent in 2009 to 18.2 percent in 2011 and then decreased to 13.1 percent in 2013 (Exhibit 3).

**EXHIBIT 3:**
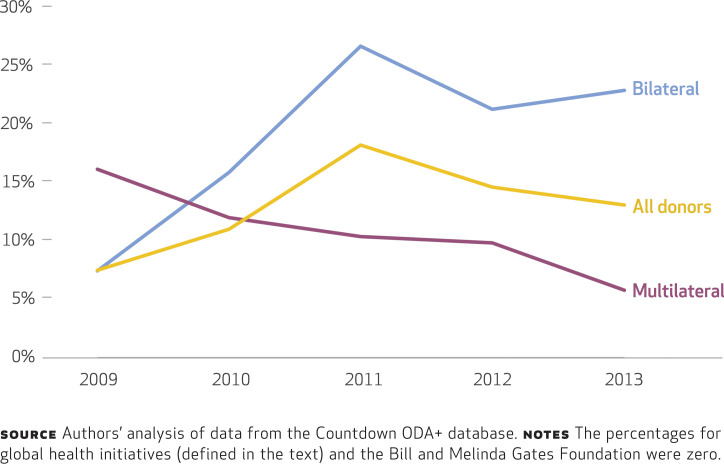
Percentages of official development assistance for reproductive, maternal, newborn, and child health delivered through government channels and disbursed as pooled funds (with those of other donors), by donor type, 2008–13

Between 2009 and 2013, disbursements of total ODA+ to reproductive, maternal, newborn, and child health through government channels increased for lower-middle- and low-income countries (from $1,403.5 million to $2,035.3 million and from $1,292.7 million to $1,785.8 million, respectively) (Exhibit 4). However, as a proportion of total ODA+ to reproductive, maternal, newborn, and child health, funds disbursed through the government decreased in both income groups (from 45.9 percent to 39.3 percent and from 47.0 percent to 36.2 percent, respectively) (Appendix Table 5).^20^ Of the funds channeled through the government, 9.6 percent were delivered as pooled funds with other donors in lower-middle-income countries, and 15.8 percent were delivered as pooled funds in low-income countries. Trends in alignment showed a high degree of heterogeneity (Appendix Figure 1).^20^ However, the top three recipients received about half of their funds for reproductive, maternal, newborn, and child health through their governments (Nigeria 46.6 percent, Ethiopia 45.9 percent, and Kenya 41.4 percent) (data not shown).

**EXHIBIT 4:**
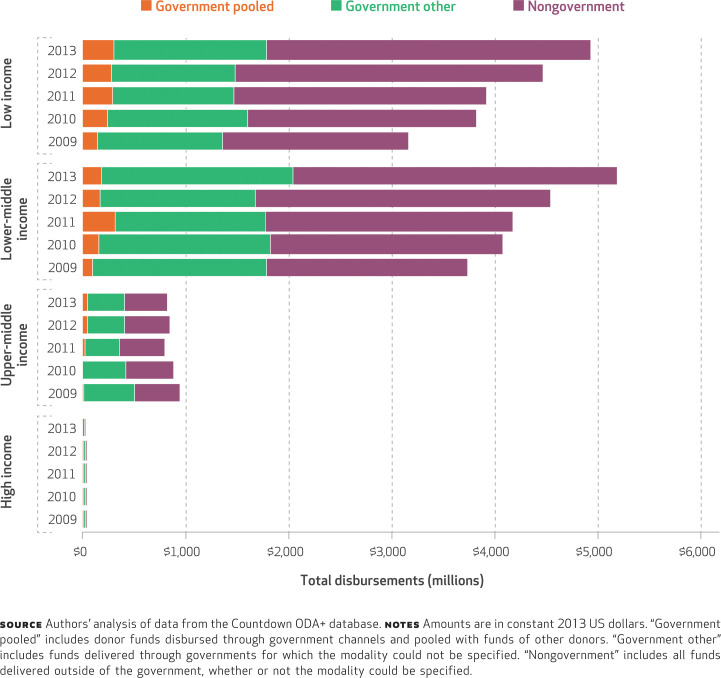
Total disbursements of official development assistance for reproductive, maternal, newborn, and child health, by channel or modality and recipient-country income group, 2009–13

Qualitative findings showed that donors’ headquarters staff members had concerns about the use of pooled funds, given the need for control and accountability to their own governments that resources are spent in appropriate ways.

One staff member said: “For us as an agency…, the solution is not, essentially, to have all the funds in one basket and then make grants to countries; we have some specific things that we need to try to accomplish.… The degree of control and accountability that we need, therefore—it’s usually not going to be satisfied by, essentially, having all of our funds in global mechanisms.”

**harmonization of donor funding** On average, donors provided ODA+ to reproductive, maternal, newborn, and child health to about forty-five recipient countries between 2006 and 2013. Seven donors donated to more than a hundred recipient countries, including three of the top ten donors (the United States donated to 110.6 countries, the Global Fund to 109.2, and the European Union to 110.7) (Appendix Table 7).^20^ For most donors, the Theil Index was less than or near 1.0, which indicates high proliferation of funding—that is, disbursement of small levels of ODA+ to reproductive, maternal, newborn, and child health to many countries. The Theil Index overall was relatively unchanged between 2006– 08 (0.95) and 2012–13 (0.99) (Appendix Figure 2).^20^ Multilaterals had higher levels of proliferation of funding, with a Theil Index of 0.57 in 2006–08 and 0.72 in 2012–13, than bilaterals (Theil Index: 1.12 and 1.11, respectively). The Gates Foundation had the lowest levels of proliferation of funding (Theil Index: 1.49 in 2009–11 and 1.91 in 2012–13) (Appendix Figure 2).^20^

In a given year in the period 2008–13, recipient countries received ODA+ to reproductive, maternal, newborn, and child health from an average of 15.4 donors, although five countries had more than 30.0 donors (Mozambique had 32.6; Tanzania and Kenya each had 31.4; and Afghanistan and Ethiopia each had 30.2) (data not shown). There was little change in the fragmentation of funding over time (the HHI was around 0.32 for the time interval averages across the time period) (Appendix Table 9).^20^ However, fragmentation was reduced for countries in sub-Saharan Africa (HHIs: 0.23 in 2006–08 and 0.26 in 2012– 13) and in Latin America and the Caribbean (HHIs: 0.39 and 0.44, respectively), whereas it increased in South Asia (HHIs: 0.19 and 0.15, respectively) (Appendix Table 8).^20^ ODA+ to reproductive, maternal, newborn, and child health was more fragmented across low-income countries, although there was substantial variation within country income groups (Appendix Figure 1c).^20^

Our qualitative research findings showed that bilateral donor representatives were especially concerned about the risk of proliferation of donors in recipient countries. Other donor types acknowledged that the risk of proliferation of donors was not explicitly considered when allocating funds to countries, with allocations based on priority areas and country proposals. Most staff members in donor headquarters who participated in our interviews agreed that fragmentation increases transaction costs for recipient governments, hinders the coordination of donors with different priorities and funding models, and risks duplication of efforts. However, one headquarters staff member did not think that “having multiple partners engaged on common issues is inherently a problem.”

**tanzania case study** Tanzania received US$2.6 billion of ODA+ to reproductive, maternal, newborn, and child health in the period 2008–13. The top ten donors accounted for 86.0 percent of all funds, with just the top two (the United States and the Global Fund) making up 55.8 percent of all of the assistance. Assistance delivered through government channels decreased from 69.9 percent of the total assistance in 2008 to 52.8 percent in 2013, and the share of funds channeled through the government but pooled with funds from other donors decreased from 2011 to 2013 (Appendix Figure 3a).^20^ There was increased reliance on project funding, from 49.0 percent of the assistance in 2008 to 90.9 percent in 2013 (Appendix Figure 3a).^20^ In the same period, the United States delivered 40.7 percent of its funds through the Tanzanian government, compared to 80.1 percent for the Global Fund (data not shown). Neither donor disbursed money as pooled funds with other donors.

ODA+ to reproductive, maternal, newborn, and child health in Tanzania was highly volatile over the period 2008–13. With the exception of the United States, disbursements from the top ten donors fluctuated considerably (Appendix Figure 3c).^20^ Fluctuations were greatest for Global Fund disbursements, which oscillated between $61.0 million and $124.9 during the period (Appendix Figure 3c).^20^

In our qualitative research, Tanzania-based respondents reported that donors had disbursed funds through the government without giving sufficient consideration to strengthening health financial management capacity. As a result, donors had been disappointed by the results obtained, which—coupled with increased pressures to “attribute [money] to results”—meant that donors had reverted to funding projects instead of using pooled approaches with other donors.

One representative of a donor said: “Everybody thought we had found the Holy Grail, but I think now the people are a little bit more critical and realize that it’s not that easy. And now we see another move—moving away from [general budget support and health-]sector budget support and back to projects.”

The average number of donors disbursing ODA+ to reproductive, maternal, newborn, and child health in Tanzania increased from twenty-eight in 2006–08 to thirty-five in 2012– 13 (Appendix Table 9).^20^ Fragmentation of donor funding increased slightly between 2009–11 (HHI: 0.21) and 2012–13 (HHI: 0.18). The assistance was delivered as 2,563 transactions in 2008, increasing to 4,258 transactions in 2011 (Appendix Figure 3b).^20^ The United States and Global Fund accounted for 161 and 11 of these transactions in 2008 and 225 and 9 transactions in 2013, respectively (data not shown).

Like donor headquarters staff members, most donor representatives interviewed in our qualitative research in Tanzania reported concerns about the levels of fragmentation of funding and its impact on the quality of the dialogue between the government and donors. One representative based in Tanzania said, “There are so many activities and initiatives and implementing agencies that the dialogue often remains very general, and at a higher level we are not able, because of the multitude of actors, to coordinate all activities very well.”

However, another interviewee perceived that fragmentation of donor funding resulted in “a more active dialogue in health,” with “more substance in the discussions between donors and government in the health sector.”

## Discussion

Our study analyzed trends in the period 2008–13 in two key principles of aid effectiveness: the alignment of donor funding with country strategies and financial management systems and the harmonization of the funding with that of other donors in relation to ODA+ to reproductive, maternal, newborn, and child health. We found little evidence of improvement in donors’ adherence to either principle overall, although we identified both improvements and deteriorations in some metrics for certain donors and recipients. Alignment of the assistance deteriorated in the study period, with most donors moving away from pooled funding. Harmonization of donor funding remained constant, despite increased funding for reproductive, maternal, newborn, and child health. High levels of fragmentation of funding at the country level remain a concern, as demonstrated by the case of Tanzania.

Achieving alignment of donor funding with country strategies requires that donors use a country’s institutional and management arrangements, which we assessed as the proportion of funds that were pooled by multiple donors and delivered through government channels. In the study period, fewer than half of all reproductive, maternal, newborn, and child health funds from donors to recipient countries were delivered through governments, and the share of donor funding to governments decreased over time across lower-middle- and low-income countries. This is surprising, given donors’ commitments to the Paris Declaration and its five principles, which call for greater alignment of donor funding with recipient countries’ priorities and systems than was typical in the past.

We found substantial variation across donor types: Multilaterals disbursed the highest proportion of funds through governments, while some of the largest bilateral donors disbursed the majority of their funds through nongovernment channels (for instance, the United States disbursed 65.4 percent of its funds this way). By pooling funds with other donors, donors could create an effective means of aid coordination, but we found that donors’ enthusiasm for pooling funds has decreased in recent years, as shown by the decrease in the share of pooled funds (from 18.2 percent in 2011 to 13.1 percent in 2013).

These trends are concerning because they represent a reversal of gains perceived by our country-level participants in increased donor coordination and greater government control of funds. While still aligned to government systems, project funding channeled by donors through governments increases transaction costs, since each project requires separate negotiation, management, and reporting.11 Projects delivered outside the government are the least coordinated with or aligned to country strategies. Results from our qualitative research showed that donor disillusionment with progress and the desires to control funds and for greater accountability to domestic populations of donor countries—to make it possible to demonstrate the effective use of funds—are making these modalities more attractive. Our results are similar to those of a study in Uganda.^14^

Funding volatility at the country level was also substantial, as seen in Tanzania. Year-by-year fluctuation of funds makes it hard for governments to plan activities and honor their commitments to their citizens.^27^ This is particularly worrisome, as the poorest countries have been shown to be more likely to receive unpredictable amounts of official development assistance.^10^

Through international agreements, donors have repeatedly committed to becoming more harmonized with other donors by increasing the concentration of funding.^2,28^ We found that the proliferation of official and Gates assistance for reproductive, maternal, newborn, and child health remained relatively constant over time. Proliferation was lowest for bilateral donors and the Gates Foundation. Multilateral donors and global health initiatives have resource-funding formulas that require them to fund all eligible countries. Therefore, we would have expected them to spread their funds more evenly, but we would have expected bilaterals to make further strides toward concentration of funding. Two studies that explored trends in overall concentration of official development assistance also reported little progress.^23,29^

**The key may be to find a balance between meeting recipients’ needs and not undermining the effectiveness of official development assistance.**

We found little change between 2008 and 2013 in fragmentation trends for ODA+ to reproductive, maternal, newborn, and child health at the country level. However, low-income countries received the most fragmented funds, with fragmentation levels falling in sub-Saharan Africa and increasing in Asia. The fact that funds became less fragmented in sub-Saharan Africa despite increases in funding and the number of donors suggests that funds were concentrated among a few donors—which is encouraging. Nevertheless, some of the poorest countries still had high levels of fragmentation in funding, as seen in Tanzania. This is consistent with findings from a study of official development assistance for health.^9^ High degrees of proliferation and fragmentation in funding decrease the effectiveness of the assistance by increasing transaction costs for the recipient government and hindering coordination with other donors,11 especially when donors are disbursing funds through their own projects outside of government channels. Arne Bigsten and Sven Tengstam attribute a lack of harmonization both to donors’ having differing goals and a tendency to micromanage developmental projects and to the possibility of some donors becoming free riders when there is harmonization of funding.^30^

## Policy Implications

The findings from this study suggest the need for reflection on the future of official development assistance to low- and middle-income countries. There have been concerns about the stagnation of donor health-sector funding,^31^ and, indeed, only one of the United Nations’ seventeen Sustainable Development Goals to be achieved by 2030 directly addresses health (compared to three of the Millennium Development Goals). However, it is not enough to advocate for increased funding, particularly if funds are delivered for separate projects by myriad donors with diverse requirements. With a higher number of goals and indicators for the Sustainable Development Goals than for the Millennium Development Goals, there is potential for increasing the fragmentation and recipient countries’ transaction costs of official development assistance. It may be unrealistic to expect UN agencies to concentrate this assistance, but they could reduce transaction costs by coordinating funds in-country—for instance, through delegated cooperation mechanisms. In addition, there is no agreed-on ideal level of fragmentation of funding, and too much concentration of funding may also be harmful.^11^ The key may be to find a balance between meeting recipients’ needs and not undermining the effectiveness of official development assistance.

Better methods are also needed to assess that effectiveness. A previous study showed that the Paris Declaration principles are broad and multidimensional, while the indicators proposed to assess progress are narrow and imprecise and rely too heavily on quantitative data.^3^ The indicators used in our study capitalize on publicly available data to compare countries globally. However, they do not capture some principles of aid effectiveness (notably country ownership of national strategies, management for results, or accountability), nor do they capture all aspects of alignment and harmonization (such as donors’ ability to fill gaps in national strategies or coordinate in-country activities). Country ownership of national strategies, management for results, and accountability are best assessed qualitatively. Therefore, there is a need for more single- or multicountry case studies, as well as better methods to assess qualitative indicators globally (for instance, surveys that assess global aid effectiveness declarations could include open-ended questions).^3^

## Conclusion

When donors are assessed for their support in implementing the Sustainable Development Goals, they should be held accountable not only for how much they disburse to achieve different goals or subgoal targets, but also for how aligned their funds are with national health plans, how the funds are disbursed, and the number of donors already operating in the health sector. Our study has shown that it is possible to monitor the alignment and harmonization of donor funds by using existing data sources and country case studies. The Global Financing Facility is a financing instrument launched at the Financing for Development Conference in Addis Ababa in 2015 in an effort to strengthen the Paris principles by improving domestic and donor resources for reproductive, maternal, newborn, child, and adolescent health in sixty-three high-burden countries. It will be important to link this initiative with processes to monitor progress toward the Sustainable Development Goals.

A previous version of this paper was presented at the Conference of the International Association for Health Economics, Milan, July 12–15, 2015. This work was funded through a subgrant from the US Fund for UNICEF under its Countdown to 2015 for Maternal, Newborn, and Child Survival Grant (No. OPP1058954) from the Bill and Melinda Gates Foundation. The authors thank the interviewees for sharing their time and expertise. This is an open access article distributed in accordance with the terms of the Creative Commons Attribution (CC BY 4.0) license, which permits others to distribute, remix, adapt, and build upon this work, for commercial use, provided the original work is properly cited. See https://creativecommons.org/licenses/by/4.0/.

## NOTES

1 Development Assistance Committee. Aid to health [Internet]. Paris: Organization for Economic Cooperation and Development; 2017 [cited 2017 Sep 7]. Available from: http://www.oecd.org/dac/stats/aidtohealth.htm

2 Organization for Economic Cooperation and Development. Paris declaration on aid effectiveness. Paris: OECD; 2005.

3 Martinez-Alvarez M. Development assistance for health in Tanzania: has the sector wide approach achieved the principles of aid effectiveness? [dissertation] [Internet]. London: London School of Hygiene and Tropical Medicine; 2014 [cited 2017 Sep 7]. Available from: http://researchonline.lshtm.ac.uk/2095790/1/2014_PHP_PhD_Martinez%20Alvarez_M_NEW.pdf

4 McCoy D, Chand S, Sridhar D. Global health funding: how much, where it comes from and where it goes. Health Policy Plan. 2009;24(6): 407–17.

8 Arregoces L, Daly F, Pitt C, Hsu J, Martinez-Alvarez M, Greco G, et al. Countdown to 2015: changes in official development assistance to reproductive, maternal, newborn, and child health, and assessment of progress between 2003 and 2012. Lancet Glob Health. 2015;3(7): e410–21.

9 Frot ESantiso J. Crushed aid: fragmentation in sectoral aid [Internet]. Rochester (NY): SSRN; 2009 Dec 3 [cited 2017 Sep 7]. (SITE Working Paper No. 6). Available for download from: https://papers.ssrn.com/sol3/papers.cfm?abstract_id=1517340

10 Celasun O, Walliser J. Predictability of aid: do fickle donors undermine aid effectiveness? Economic Policy. 2008(55):545–86, 590–94.

11 Acharya A, Fuzzo de Lima AT, Moore MP. Proliferation and fragmentation: transactions costs and the value of aid. J Dev Stud. 2006;42(1):1–21.

12 International Health Partnership and Related Initiatives. Progress in the international health partnership & related initiatives (IHP+): 2014 performance report [Internet]. Geneva: IHP+; [cited 2017 Sep 7]. Available from: https://www.internationalhealthpartnership.net/fileadmin/uploads/ihp/Result_2014/Documents/IHP_report-ENG-WEB_v2.PDF

13 UNICEF. Progress for children: achieving the MDGs with equity [Internet]. New York (NY): UNICEF; 2010 Sep [cited 2017 Sep 7]. Available from: https://www.unicef.org/publications/files/Progress_for_Children-No.9_EN_081710.pdf

14 Stierman E, Ssengooba F, Bennett S. Aid alignment: a longer term lens on trends in development assistance for health in Uganda. Global Health. 2013;9(7):7.

15 Oliveira Cruz V. Agency theory and the relationship between national governments and development partners—case study of Uganda. London: London School of Hygiene and Tropical Medicine; 2007.

16 Delph E. Global vertical programmes: the future of aid or a quick fix? Quezon City (Philippines): EURODAD; 2008.

17 Dijkstra G. The new aid paradigm: a case of policy incoherence [Internet]. New York (NY): United Nations, Department of Economic and Social Affairs; 2013 Dec [cited 2017 Sep 7]. (DESA Working Paper No. 128). Available from: http://www.un.org/esa/desa/papers/2013/wp128_2013.pdf

18 Grollman C, Arregoces L, Martínez-Álvarez M, Pitt C, Mills A, Borghi J. 11 years of tracking aid to reproductive, maternal, newborn, and child health: estimates and analysis for 2003–13 from the Countdown to 2015. Lancet Glob Health. 2017;5(1): e104–14.

19 Grollman C, Arregoces L, Martinez-Alvarez M, Pitt C, Powell-Jackson T, Hsu J, et al. Developing a dataset to track aid for reproductive, maternal, newborn and child health, 2003– 2013. Sci Data. 2017;4:170038.

20 To access the Appendix, click on the Details tab of the article online.

21 Organization for Economic Cooperation and Development. The Busan Partnership for Effective Development Co-operation [Internet]. Paris: OECD; c 2017 [cited 2017 Sep 7]. Available from: http://www.oecd.org/development/effectiveness/busanpartnership.htm

22 The term government channels refers to government financial management systems. Official development assistance can be disbursed through these channels or by private and nonprofit organizations. Pooled modalities include general or health-sector budget support and health basket funds (explained below). Disbursing funds through pooled modalities gives recipient governments greater control over how resources are used and accounted for. Health basket funds are donor funds for health that are pooled and then allocated to recipients (usually through government channels). Governments and donors jointly agree on how the funds are to be used and accounted for.

23 Brown S, Swiss L. The hollow ring of donor commitment: country concentration and the decoupling of aid-effectiveness norms from donor practice. Dev Policy Rev. 2013;31(6): 737–55.

24 World Bank. World Bank country and lending groups [Internet]. Washington (DC): World Bank; [cited 2017 Oct 5]. Available from: https://datahelpdesk.worldbank.org/knowledgebase/articles/906519-world-bank-country-and-lendinggroups

25 Vassall A, Shotton J, Reshetnyk OK, Hasanaj-Goossens L,Weil O, Vohra J, et al. Tracking aid flows for development assistance for health. Glob Health Action. 2014;7:23510.

26 Braun V, Clarke V. Using thematic analysis in psychology. Qual Res Psychol. 2006;3(2):77–101.

27 Juliet NO, Freddie S, Okuonzi S. Can donor aid for health be effective in a poor country? Assessment of prerequisites for aid effectiveness in Uganda. Pan Afr Med J. 2009;3(2):9.

28 Organization for Economic Cooperation and Development. Rome declaration on harmonisation [Internet]. Paris: OECD; 2003 [cited 2017 Sep 7]. Available from: https://www.oecd.org/dac/effectiveness/31451637.pdf

29 Aldasoro I, Nunnenkamp P, Thiele R. Less aid proliferation and more donor coordination? The wide gap between words and deeds. J Int Dev. 2010;22(7):920–40.

30 Bigsten A, Tengstam S. International coordination and the effectiveness of aid. World Dev. 2015;69:75–85.

31 Dieleman J, Murray CJL, Haakenstad A. Financing global health 2015: development assistance steady on the path to new Global Goals [Internet]. Seattle (WA): Institute for Health Metrics and Evaluation; 2016 [cited 2017 Sep 7]. Available from: http://www.healthdata.org/sites/default/files/files/policy_report/FGH/2016/IHME_PolicyReport_FGH_2015.pdf

